# Musculoskeletal discomfort associated with special operations uniform use in elite police officers: a cross-sectional study

**DOI:** 10.3389/fpubh.2026.1817387

**Published:** 2026-05-14

**Authors:** Camila B. B. Silva, Paulo Vinicios C. Zovico, Geanderson S. Oliveira, Pedro F. C Fortes Junior, Matheus Florindo de Deus, Michell V. Viana, Valentina Bullo, Marco Bergamin, Stefano Gobbo, Roberta L. Rica, Danilo Sales Bocalini

**Affiliations:** 1 Experimental Physiology and Biochemistry Laboratory, Physical Education Sport Center, Federal University of Espirito Santo, Vitoria, Espirito Santo, Brazil; 2 Department of Medicine, University of Padova, Padova, Italy; 3 Faculty of Physical Education, University Center Estacio Vitoria, Vitoria, Espirito Santo, Brazil

**Keywords:** operational performance, performance, policing, public security, security

## Abstract

**Background:**

Personal protective equipment (PPE) and operational gear are mandatory for tactical police duties. However, due to their physical weight and load burden, they can promote musculoskeletal discomfort (MD).

**Objective:**

The study aimed to investigate MD among police officers from a special operations company (SOC), with and without the use of a special operations uniform (SOU).

**Methods:**

A total of 42 police officers from the special operations company voluntarily participated in this study. MD was assessed using a standardized body diagram under two conditions: With and without the SOU. The instrument divided the body into 27 regions and provided each participant with a discomfort index on a scale ranging from 1 to 5. In addition, physical activity levels and anthropometric characteristics were measured and correlated with overall discomfort and service time.

**Results:**

The overall mean age was 37.10 ± 6.05 years, with a body mass index (BMI) of 28.20 ± 3.60 kg/m^2^ and an average service time of 13.00 ± 6.70 years. A significant difference (*p* < 0.05) was observed in body mass between officers wearing the SOU (100.40 ± 12.24 kg) and those not wearing it (86.80 ± 11.74 kg). The use of the SOU was associated with an increase in MD compared to the no-uniform condition. General discomfort correlated with waist circumference (WC; *p* = 0.0329) and the waist-to-height ratio (WHtR*; p* = 0.0203). Service time showed a positive correlation with the waist-to-hip ratio (WHR) (*p* = 0.0120) and the behavioral domain (*p* = 0.0015).

**Conclusion:**

The use of the tactical police uniform causes muscle discomfort among police officers from a special operations company.

## 1 Introduction

During ostensive policing, military police officers (MPOs) are easily identified by their uniforms, as well as by the appropriate security equipment, weapons, and means of transport for each activity. However, MPOs face intense exposure to occupational demands due to work schedules that may extend up to 12 h, followed by 36-h rest periods, thereby increasing occupational risks. This exposure goes beyond the time spent on external patrols and includes prolonged periods seated in vehicles in non-ergonomic postures. This can result in musculoskeletal overload, which may be worsened by the continuous use of personal protective equipment (PPE), essential for protecting officers against potential chemical, physical, or biological risks during their duties (1, 2).

Traditional Brazilian PPE includes a variety of elements, such as ballistic vests, tactical belts, leg holsters, weapons of different types (short, medium, and non-lethal) with magazines, knives, boots, handcuffs, communication radios, and flashlights. These items can be arranged in different ways, including attachment to vests, belts, legs, or combinations of these. However, the amount of this equipment increases when specialized police officers are on standby, including shields, helmets, gas masks, knee pads, and elbow pads. Studies have shown that army soldiers, military special forces, elite tactical units, and general duty police carry loads of approximately 48 kg (3), 55 kg (4), 22 kg–40 kg (5, 6), and 10 kg (7), respectively. In Brazil, Manzolli et al. (8) showed that police officers from the mounted police regiment may carry a load of approximately 15 kg in PPE.

In this context, previous evidence suggests that the use of personal protective equipment (PPE) may increase discomfort and perceived exertion during physical tasks, as well as elevate heart rate and metabolic energy expenditure, particularly as the load increases (7, 9). Furthermore, heavier PPE has been associated with reductions in neuromuscular performance, although certain task-specific skills, such as marksmanship, may remain unaffected (5). In addition, PPE may restrict joint range of motion, with the design and distribution of the equipment exerting a greater influence on movement limitation than the total load itself (9, 10). Importantly, sex-specific responses to PPE, including differences in perceived discomfort and physiological strain, have also been reported and should be considered when interpreting these findings.

In addition, the combination of PPE can negatively impact musculoskeletal health (11) and reduce professionals’ occupational capacity (12–15). Thus, we suggest that injuries and muscle discomfort among police officers may be associated with the combined effects of PPE use and factors such as extended working hours, insufficient physical activity, and lack of proper planning. According to Grani (16), records from the Medical Board of the Police Hospital from March 2017 to March 2018 indicated that 1,582 medical certificates (16% of the total) were related to musculoskeletal pain and injuries among police officers in Paraná, Brazil. Larsen et al. (17) reported that 41.3% of Swedish police officers experienced musculoskeletal pain at least once a week in the previous 3 months. An association between discomfort from wearing mandatory equipment and musculoskeletal pain has been identified, particularly in relation to duty belts and body armor (17). Therefore, it is essential to know the conditions of exposure to risks and the specific impacts that musculoskeletal discomfort (MD) and lifestyle factors may have on the lives of these workers, so that intervention strategies and occupational health protection measures can be planned and carried out in an appropriate manner. However, it is important to emphasize that, although clinical practice and ergonomics have established the analysis of musculoskeletal discomfort as an area of intervention, conclusive evidence regarding police officers remains lacking.

Considering the findings reported by Hudson et al. (9), previous studies have primarily focused on general duty or conventional tactical police populations, often using observational designs that do not allow direct comparisons between equipment conditions. Moreover, although the association between PPE and musculoskeletal discomfort has been previously described (11), these investigations have not specifically examined elite special operations units under controlled within-subject conditions. Importantly, specialized police units are exposed to distinct operational demands, including higher equipment loads, more complex tactical tasks, and prolonged use of full protective gear, which may result in different ergonomic and physiological responses compared to conventional police forces. In addition, there is a scarcity of data from Latin American contexts, particularly involving elite units, limiting the external validity of findings derived from studies conducted in North America and Europe.

Therefore, the present study aimed to evaluate musculoskeletal discomfort in officers from a special operations company (SOC) of the Espírito Santo Special Missions Battalion (SMB), comparing two conditions—with and without the use of the special operations uniform—using a within-subject design. Thus, the novelty of this study lies in (i) the inclusion of an elite tactical population, (ii) the use of a within-subject comparison between operational conditions, and (iii) the investigation of this phenomenon in a Latin American context. We hypothesized that the use of the special operations uniform (SOU) would be associated with increased musculoskeletal discomfort among these professionals.

## 2 Materials and methods

### 2.1 Study design

This study was designed as a descriptive, cross-sectional, analytical investigation with a repeated-measures component, conducted using questionnaire-based instruments and anthropometric assessments. Participants were recruited from the Espírito Santo Special Missions Battalion (SMB), Brazil, through direct contact with the researchers, as well as verbal and digital dissemination strategies.

The study protocol was approved by the Research Ethics Committee of the Federal University of Espírito Santo (protocol number: 6.275.609/2023). All participants voluntarily agreed to participate and provided written informed consent in accordance with Resolution no. 466/2012 of the Brazilian National Health Council and the Declaration of Helsinki.

A within-subject design was adopted, in which each participant was evaluated under two conditions. The order of assessments was randomized using a block randomization procedure with blocks of six participants to minimize potential order effects.

For statistical analysis, corrections for multiple comparisons were performed using the false discovery rate (FDR), and effect sizes were calculated to complement inferential statistics.

The SMB is an elite unit of the Espírito Santo State Military Police, specializing in high-complexity operations requiring advanced tactical expertise. These include civil disturbance control, operations involving explosive devices, hostage situations, and other critical incidents.

The unit currently comprises approximately 188 officers and is considered a specialized response force within the state police structure. Its operational doctrine emphasizes advanced technical training, continuous physical preparation, discipline, and commitment to duty.

In general, SMB officers are trained to operate in a wide range of scenarios, including explosive ordnance disposal, chemical incident response, high-angle and aquatic rescue operations, prison interventions, tactical entry operations, crisis management, hostage negotiation, and precision shooting.

### 2.2 Sample

Initially, police officers volunteered to participate in the study. After applying the eligibility criteria, 42 officers from the SOC were included. The inclusion criteria were as follows: (i) active duty military police officers, (ii) age between 25 and 50 years, and (iii) a minimum of 1 year of operational service. Years of service were recorded and analyzed as a continuous variable but were not used as an inclusion criterion. The exclusion criteria included officers who were temporarily removed from duty due to medical or psychological conditions, those who did not sign the informed consent form, and those who did not complete all study procedures. Thus, the sample represented approximately 22% of the SMB and 76% of the SOC population.

### 2.3 Experimental design

Data were collected in participants’ work environment during their regular duty shifts. The total duration of data collection was approximately 3 weeks, according to service orders. Each participant completed all assessments in a single session lasting approximately 30 min. All participants received standardized instructions before data collection. The assessment sequence included the following: Informed consent, lifestyle evaluation (Fantastic Lifestyle questionnaire), physical activity level (International Physical Activity Questionnaire (IPAQ)), anthropometric assessment, and musculoskeletal discomfort evaluation under two conditions.

**Figure 1:**
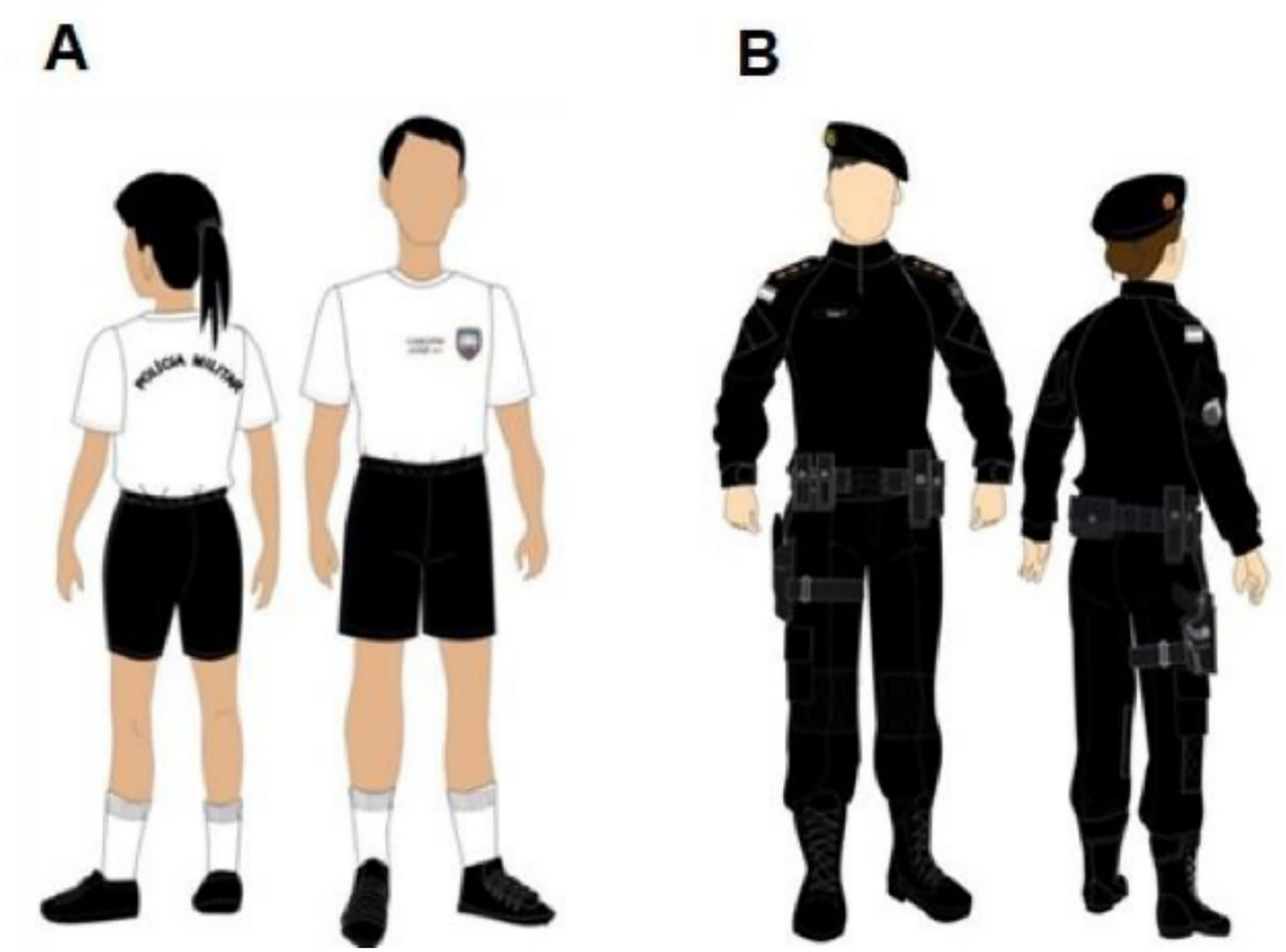
Models of SOC (special operations company) uniforms without PPE. **(A)** Physical training uniform (PTU). Reproduced with permission from “Physical Education Uniform”, Military Police of Espírito Santo (PMES) (https://pm.es.gov.br/). **(B)** Special operations uniform (SOU). Reproduced with permission from “Special Operations Uniform”, Military Police of Espírito Santo (PMES) (https://pm.es.gov.br/).

A within-subject design was adopted, in which each participant was evaluated under both conditions: Physical training uniform (PTU) and SOU with PPE (Figure 1). The order of conditions was randomized to minimize potential order effects. The SOU included mandatory equipment such as ballistic vests, tactical belts, holsters, weapons, and communication devices.

To evaluate musculoskeletal discomfort, the PTU and SOU conditions were randomized using a randomization website with blocks of six participants per condition. Each block allocated two conditions per evaluation, ensuring balanced allocation in the study and promoting similarity in initial measurements between the conditions. This strategy was used to reduce the risk of bias and is considered a quality criterion in experimental designs investigating comparisons between conditions.

Standardized cutoff values were applied where appropriate. Body mass index (BMI) was classified according to established guidelines, and a waist-to-height ratio (WHtR) of ≥0.50 was used to indicate increased health risk. Participants were classified as physically active if they accumulated at least 150 min per week of physical activity, according to World Health Organization (WHO) recommendations.

### 2.4 Evaluated parameters

#### 2.4.1 Anthropometric evaluation

To perform the anthropometric assessment, body mass (MarteScientific digital scale, L200, SP) was measured to a precision of 0.1 kg and height (Cardiomed Stadiometer model WCS) to a precision of 0.1 cm. Body mass index (BMI) was obtained using the following equation: BMI = body mass ÷ height^2^. The data were classified according to Nutall et al. (17) as normal weight (18.5 ≤ 25.0 kg/m^2^), overweight (25.0 ≤ 30.0 kg/m^2^), and obesity (30.0 ≤ 40.0 kg/m^2^).

Waist circumference (WC) was measured using an inelastic tape at the midpoint between the lower border of the last rib and the upper border of the iliac crest, following standard procedures. The criteria used to classify cardiovascular risk were based on previous studies (18, 19): Increased risk (men: ≥94 cm, women: ≥80 cm) and substantially increased risk (men: ≥102 cm, women: ≥88 cm).

The waist-to-height ratio (WHtR) was calculated by dividing WC by height (both in centimeters–cm), yielding a maximum possible value of 1. A cutoff point of 0.50 was used to classify low and increased health risk, as in previous studies (20, 21).

Body composition was estimated using a doubly indirect method based on the measurement of biceps, triceps, subscapular, and suprailiac skinfolds (Mitutoyo Cescorf Plicometer, Porto Alegre, Brazil). Body fat percentage (F%) was estimated using the equation proposed by Durnin and Wormersley and classified as normal (<18%), overweight (18–24.9%), and obesity (>25%), according to previous studies from our group (21).

#### 2.4.2 Physical activity

Participants’ physical activity levels were assessed using the International Physical Activity Questionnaire (IPAQ), according to previous studies (21–24). The questionnaire evaluates weekly physical activity based on frequency and duration, including walking, moderate, and vigorous activities. Physical activity levels were classified using standardized cutoff values based on World Health Organization (WHO) guidelines. Participants were considered physically active if they accumulated at least 150 min per week of moderate-intensity physical activity or an equivalent combination of moderate and vigorous activity. Those who did not meet this threshold were classified as physically inactive. This classification approach has been widely used in epidemiological and occupational health studies.

#### 2.4.3 Lifestyle

Lifestyle was assessed using the Fantastic Lifestyle Questionnaire, previously validated for the Brazilian population (25) and widely applied in studies conducted by our group (8, 22, 23). This self-administered instrument evaluates behaviors over the previous month across 25 items distributed into nine domains: Family/friends, physical activity, nutrition, tobacco/toxics, alcohol, sleep/seat belt/stress/safe sex, behavior type, insight, and career. The questionnaire consists of Likert-scale responses (23 items with five response options and two dichotomous items), yielding a total score ranging from 0 to 100 points. Standardized cutoff values were applied to classify lifestyle categories based on the total score. Participants were categorized as follows: Excellent (85–100 points), very good (70–84 points), good (55–69 points), fair (35–54 points), and needs improvement (0–34 points). This classification system has been previously validated and is commonly used in epidemiological and occupational health research.

#### 2.4.4 Musculoskeletal discomfort

Musculoskeletal discomfort was assessed using a body diagram according to Corlett and Manenica (26), previously used by our group (8, 23). This instrument is designed to evaluate painful areas by dividing the body into 27 parts and provides the respondent with a pain index on a scale ranging from 1 (no pain) to 5 (extreme pain). This instrument allows responses regarding the presence, location, and intensity of pain. To assess overall reported discomfort (general discomfort), the sum of the 27 body segments was calculated. Musculoskeletal discomfort was classified into the following categories: 1 (no pain), 2 (mild pain), 3 (moderate pain), 4 (severe pain), and 5 (extreme pain).

All variables were classified using standardized and previously validated cutoff values, ensuring consistency with epidemiological and occupational health research guidelines, according to a previous study by our group (8, 23).

#### 2.4.5 Statistical analysis

Data are presented as absolute (*n*) and relative (%) frequencies for qualitative variables and as means ± standard deviations, the coefficient of variation (CV), differences in means (DM), and 95% confidence intervals (95% CIs) for quantitative variables, when appropriate. After analyzing data normality using the D’Agostino-Pearson test, participants’ performance under the PTU and SOU conditions was compared using the paired *t*-test. Pearson’s correlation test was used to determine the strength and direction of the correlation between anthropometric parameters, weekly physical activity time, lifestyle, general discomfort, and length of service. The strength of the correlation was classified as weak (r = 0.10 to 0.30), moderate (r = 0.40 to 0.60), and strong (r = 0.70 to 1.00). The effect size was estimated using Hedges’ g and interpreted as small (from 0.2 to 0.5), moderate (from 0.5 to 0.8), and large (above 0.8). GraphPad Prism, version 6.00 for Windows (GraphPad Software, La Jolla, California, USA), was used for analyses, and a significance level of *p* < 0.05 was adopted.

## 3 Results

Among the participants, eight (26.2%), 23 (54.8%), and 11 (19.0%) were soldiers, corporals, and sergeants, respectively, affiliated with the SOC/SMB. Their mean age was 37.10 ± 6.50 years, with an average service duration of 13.30 ± 6.70 years.

Waist circumference (WC) risk classification indicated that 78.6% of participants were within normal limits, 16.6% were at risk, and 4.8% presented high risk. Regarding the waist-to-hip ratio (WHR), 19.0% of participants were classified as low risk, 76.2% as moderate risk, and 4.8% as high risk, with no participants classified in the very high-risk category. The mean body mass index (BMI) was.

28.20 ± 3.60 kg/m^2^, indicating an overweight status, whereas the mean body fat percentage was.

15.20 ± 4.70%, which was deemed appropriate for the cohort’s age range.

Concerning physical activity (Table 1), participants reported an average of 273.2 ± 307.9 min per week of walking and 289.9 ± 282.7 min per week of vigorous activity. The total mean weekly duration of physical activity was 875.2 ± 662.8 min, classifying the participants as physically active.

**Table 1:** General characteristics of special operations company police officers from the Espírito Santo Special Missions Battalion.

Parameters	Mean ± SD	CV
Age (years)	37.1 ± 6.5	17.0%
Service time (years)	13.3 ± 6.7	50.1%
Anthropometric characteristics		
Body mass (Kg)	86.4 ± 11.7	13.6%
Height (m)	1.75 ± 0.07	4.3%
Waist circumference (cm)	89.3 ± 7.6	8.5%
Hip circumference (cm)	102.4 ± 5.6	5.5%
WHR	0.87 ± 0.04	5.1%
∑ SF	102.2 ± 37.9	37.2%
Body fat (%)	15.2 ± 4.7	30.8%
Fat mass (Kg)	13.4 ± 5.3	39.5%
Fat-free mass (kg)	73.0 ± 8.9	12.2%
BMI (kg/m^2^)	28.2 ± 3.6	12.7%
Physical activity		
Walking time (min/week)	273.2 ± 307.9	112.7%
Moderate activity (min/week)	312.0 ± 292.4	93.7%
Vigorous activity (min/week)	289.9 ± 282.7	97.5%
Total time of physical activity (min/week)	875.2 ± 662.8	75.7%
Lifestyle		
Family/friends	6.8 ± 1.5	22.1%
Activity	5.4 ± 2	37.5%
Nutrition	5.2 ± 3.1	59.3%
Tobacco/toxics	13.1 ± 2.1	16.1%
Alcohol	9.3 ± 2.7	28.7%
Sleep	13.6 ± 3.2	23.8%
Behavior	4.1 ± 1.7	41.7%
Insight	8.7 ± 2.2	25.3%
Career	2.9 ± 0.8	27.9%
Total score	69.2 ± 10.3	14.9%

	*N*	%
Lifestyle classification		
Excellent	1	2.4%
Very good	18	42.9%
Good	20	47.6%
Fair	3	7.1%
Needs to improve	0	0.00%

Values are shown as mean ± standard deviation.

CV, Coefficient of variation; WHR, Waist-to-hip ratio; WHtR, Waist-to-height ratio; ∑SF, Sum of skinfolds; BMI, Body mass index.

Table 2 describes the quantitative data corresponding to the indicators of participants’ lifestyle and their classification. Among the lifestyle parameters, the lowest value corresponded to career (2.9 ± 0.8), while the highest value was observed for sleep (13.6 ± 3.2), resulting in a total score of 69.2 ± 10.3. Regarding the classification of lifestyle (Table 3), this study classified 2.4% of volunteers as excellent, 42.9% as very good, 47.6% as good, 7.1% as fair, and none as needing improvement.

**Table 2:** Perceived musculoskeletal discomfort in special operations company police officers from the Espírito Santo Special Missions Battalion wearing physical training and ostensive policing uniforms.

Body region	Side	Physical training uniform	Special operations uniform	MD (95%CI)	ES	P
Neck		1.43 ± 0.77	2.17 ± 1.21	34.4	Large	<0.0001
Upper Back		1.41 ± 0.77	2.12 ± 1.33	33.5	Large	<0.0001
Middle		1.38 ± 0.76	2.00 ± 1.25	31.0	Large	=0.0002
Back						
Lower back		1.83 ± 1.17	2.70 ± 1.30	32.2	Large	<0.0001
Pelvis		1.33 ± 0.90	1.79 ± 1.22	25.7	Large	=0.0001
Shoulders	Left side	1.43 ± 0.89	2.12 ± 1.41	32.5	Large	=0.0005
	Right side	1.45 ± 0.94	2.02 ± 1.33	28.2	Large	=0.0046
Arms	Left side	1.17 ± 0.58	1.79 ± 1.10	34.6	Large	=0.0010
	Right side	1.26 ± 0.83	1.67 ± 0.98	24.6	Large	=0.0266
Forearms	Left side	1.05 ± 0.22	1.43 ± 0.80	26.6	Large	=0.0034
	Right side	1.07 ± 0.34	1.38 ± 0.88	22.5	Large	=0.0039
Fists	Left side	1.12 ± 0.55	1.41 ± 0.77	20.6	Large	=0.0039
	Right side	1.12 ± 0.55	1.41 ± 0.83	20.6	Large	=0.0078
Hands	Left side	1.07 ± 0.34	1.26 ± 0.50	15.1	Large	=0.0215
	Right side	1.09 ± 0.43	1.24 ± 0.53	12.1	Large	=0.0313
Thighs	Left side	1.17 ± 0.44	1.55 ± 0.80	24.5	Large	=0.0056
	Right side	1.21 ± 0.52	1.50 ± 0.74	19.3	Large	=0.0264
Legs	Left side	1.41 ± 0.77	1.90 ± 1.03	25.8	Large	=0.0005
	Right side	1.48 ± 0.86	1.74 ± 0.91	14.9	Large	=0.0474
Ankles and feet	Left side	1.29 ± 0.71	1.79 ± 1.10	27.9	Large	=0.0033
	Right side	1.31 ± 0.78	1.70 ± 1.12	22.9	Large	=0.0244
General discomfort		27.1 ± 7.69	36.64 ± 14.52	26.0	Large	<0.0001

Values are shown as mean ± standard deviation. MD: mean difference, 95%CI: confidence interval. ES: effect size.

**Table 3:** Classification of perceived musculoskeletal discomfort in special operations company police officers from the Espírito Santo Special Missions Battalion wearing physical training and ostensive policing uniforms.

Parameters	Physical training uniform					Special operations uniform			
	Classification of musculoskeletal discomfort					Classification of musculoskeletal discomfort			
	None	Some	Moderate	Great	Extreme	None	Some	Moderate	Great
	*n* (%)	*n* (%)	*n* (%)	*n* (%)	*n* (%)	*n* (%)	*n* (%)	*n* (%)	*n* (%)
Neck	30 (71.4)	7 (16.7)	4 (9.5)	1 (2.4)	0 (0)	19 (45.3)	10 (23.8)	7 (16.7)	4 (9.5)
Upper back	31 (73.8)	6 (14.3)	4 (9.5)	1 (2.4)	0 (0)	18 (42.8)	10 (23.8)	6 (14.3)	7 (16.7)
Middle back	32 (76.2)	5 (11.9)	4 (9.5)	1 (2.4)	0 (0)	20 (47.6)	9 (21.4)	7 (16.7)	6 (14.3)
Lower back	26 (61.9)	5 (11.9)	7 (16.7)	3 (7.1)	1 (2.4)	14 (33.3)	3 (7.1)	14 (33.3)	11 (26.3)
Pelvis	37 (88.1)	1 (2.4)	3 (7.1)	0 (0)	1 (2.4)	28 (66.7)	5 (11.9)	2 (4.7)	7 (16.7)
Left side	33 (78.6)	5 (11.9)	2 (4.8)	2 (4.7)	0 (0)	22 (52.4)	9 (21.4)	4 (9.5)	7 (16.7)
Shoulders									
Right side	31 (73.8)	7 (16.7)	1 (2.4)	2 (4.7)	1 (2.4)	23 (54.8)	9 (21.4)	6 (14.3)	4 (9.5)
Left side	38 (90.4)	2 (4.8)	1 (2.4)	1 (2.4)	0 (0)	27 (64.3)	3 (7.1)	4 (9.5)	7 (16.7)
Arms									
Right side	37 (88.1)	2 (4.8)	1 (2.4)	1 (2.4)	1 (2.4)	27 (64.3)	4 (9.5)	7 (16.7)	4 (9.5)
Left side	40 (95.2)	2 (4.8)	0 (0)	0 (0)	0 (0)	33 (78.6)	1 (2.4)	3 (7.1)	4 (9.5)
Forearms									
Right side	40 (95.2)	1 (2.4)	0 (0)	0 (0)	1 (2.4)	35 (83.3)	1 (2.4)	3 (7.1)	2 (4.7)
Left side	40 (95.2)	0 (0)	1 (2.4)	1 (2.4)	0 (0)	29 (69.1)	5 (11.9)	4 (9.5)	3 (7.1)
Fists									
Right side	40 (95.2)	0 (0)	1 (2.4)	1 (2.4)	0 (0)	31 (73.8)	4 (9.5)	4 (9.5)	3 (7.1)
Left side	40 (95.2)	1 (2.4)	1 (2.4)	0 (0)	0 (0)	30 (71.4)	9 (21.4)	2 (4.7)	0 (0)
Hands									
Right side	40 (95.2)	0 (0)	2 (4.8)	0 (0)	0 (0)	31 (73.8)	7 (16.7)	4 (9.5)	0 (0)
Left side	36 (85.7)	5 (11.9)	1 (2.4)	0 (0)	0 (0)	23 (54.8)	9 (21.4)	7 (16.7)	2 (4.7)
Thighs									
Right side	35 (83.3)	5 (11.9)	2 (4.8)	0 (0)	0 (0)	24 (57.2)	8 (19.0)	9 (21.4)	1 (2.4)
Left side	32 (76.2)	6 (14.3)	4 (9.5)	0 (0)	0 (0)	18 (42.8)	9 (21.4)	10 (23.8)	4 (9.5)
Legs									
Right side	31 (73.8)	6 (14.3)	4 (9.5)	1 (2.4)	0 (0)	19 (45.3)	8 (19.0)	10 (23.8)	5 (11.9)
Left side	35 (83.3)	3 (7.1)	3 (7.1)	1 (2.4)	0 (0)	22 (52.4)	7 (16.7)	7 (16.7)	5 (11.9)
Ankles and feet									
Right side	35 (83.3)	3 (7.1)	2 (4.8)	2 (4.8)	0 (0)	25 (59.5)	4 (9.5)	7 (16.7)	6 (14.3)

**Figure 2:**
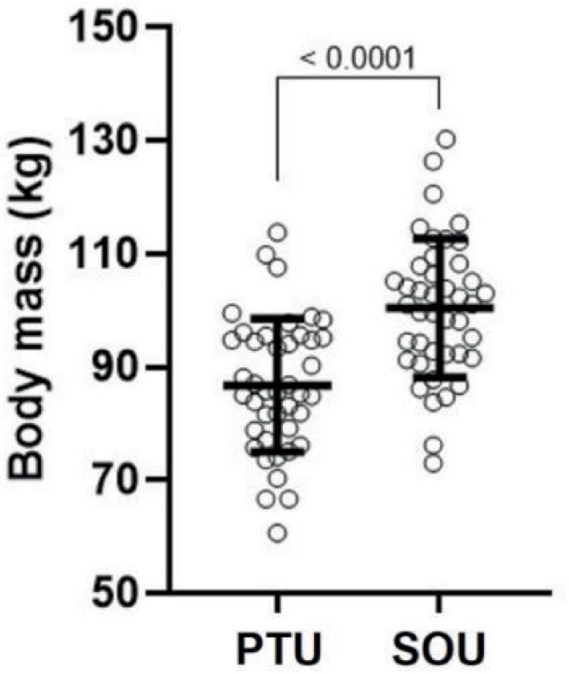
Values are shown as mean ± standard deviation of body mass in special operations company police officers from the Espírito Santo Special Missions Battalion wearing the physical training uniform (PTU) and the SOU.

Figure 2 illustrates body mass with and without the uniform. Wearing the SOU significantly increased body mass by 15.67%, from 86.80 ± 11.74 kg (PTU) to 100.4 ± 12.24 kg (SOU), with a mean difference of 13.63 kg (95% CI: 12.69–14.57; *p* < 0.0001).

Table 2 details the prevalence and magnitude of musculoskeletal discomfort. The use of uniforms (PTU vs. SOU) produced statistically significant increases in discomfort across all evaluated body segments (Table 2). As shown in Table 3, discomfort increased by 45.3% in the neck, upper, middle, and lower back, and both shoulders when using the SOU. Pelvic discomfort increased by 33.9%, upper limb discomfort by 29.3%, and lower limb discomfort by 29.4%, respectively, while general discomfort increased by 35.4% when wearing the SOU compared to the PTU.

Table 4 presents correlations between general discomfort in the SOU condition and anthropometric and lifestyle variables. General discomfort correlated weakly but significantly with waist circumference (r = −0.3298, *p* = 0.0329) and the waist-to-height ratio (WHtR: r = −0.3569, *p* = 0.0203). No significant associations were observed with other anthropometric or lifestyle measures. Furthermore, years of service showed a weak positive correlation with both length of service in the position and the waist-to-hip ratio (WHR: r = 0.3840, *p* = 0.0120), as well as a moderate correlation with the behavioral domain (r = 0.4753, *p* = 0.0015). No other significant relationships were noted.

**Table 4:** Correlation of anthropometric parameters and lifestyle variables with perceived general discomfort and service time in special operations company police officers from the Espírito Santo Special Missions Battalion wearing the SOU.

Parameters	Special operations uniform					
	General discomfort			Service time (years)		
	r	p	95%CI	r	p	95%CI
Service time (years)	−	−	−	−	−	−
General discomfort	−	−	−	−0.1941	0.2181	−0.4775 to 0.1259
Anthropometric characteristics						
Body mass (Kg)	−0.1663	0.2926	−0.4550 to 0.1540	0.0593	0.7091	−0.2578 to 0.3649
Height (m)	−0.0157	0.9213	−0.3265 to 0.2981	0.0862	0.5871	−0.2324 to 0.3881
Waist Circumference (cm)	−0.3298	0.0329	−0.5822 to −0.0194	0.2242	0.1534	−0.09475 to 0.501
Hip circumference (cm)	−0.2769	0.0758	−0.5423 to 0.0387	0.0337	0.8322	−0.2816 to 0.3424
WHR	−0.2559	0.1020	−0.5261 to 0.0613	0.3840	0.0120	0.08148–0.6218
WHtR	−0.3569	0.0203	−0.6021 to 0.0502	0.2145	0.1725	−0.1049 to 0.4937
∑ SF	−0.0400	0.8014	−0.3480 to 0.2758	0.0493	0.7567	−0.2672 to 0.3561
% Body fat	−0.0415	0.7943	−0.3493 to 0.2744	0.1592	0.3139	−0.1611 to 0.4492
Fat mass (Kg)	−0.1193	0.4519	−0.4161 to 0.2005	0.1769	0.2623	−0.1433 to 0.4636
Fat-free mass (kg)	−0.1719	0.2763	−0.4596 to 0.1484	0.0041	0.9796	−0.3086 to 0.3160
BMI (kg/m^2^)	−0.2672	0.0872	−0.5349 to 0.0492	0.0002	0.9988	−0.3121 to 0.3125
Physical activity						
Walking time (min/week)	−0.0778	0.6245	−0.3808 to 0.2404	0.2489	0.1119	−0.06874 to 0.520
Moderate activity (min/week)	−0.0119	0.9403	−0.3230 to 0.3015	−0.0487	0.7595	−0.3556 to 0.2677
Vigorous activity (min/week)	0.1418	0.3705	−0.1785 to 0.4348	0.0294	0.8534	−0.2856 to 0.3386
Total time of physical activity (min/week)	0.0456	0.7743	−0.2706 to 0.3529	0.0953	0.5482	−0.2237 to 0.3959
Lifestyle						
Family	0.0874	0.5822	−0.2313 to 0.3891	−0.1486	0.3476	−0.4405 to 0.1717
Activity	−0.2623	0.0933	−0.5311 to 0.0544	−0.1088	0.4929	−0.4073 to 0.2107
Nutrition	0.0443	0.7804	−0.2718 to 0.3518	−0.0250	0.8751	−0.3347 to 0.2896
Smoking	−0.0827	0.6028	−0.3850 to 0.2358	−0.1668	0.2910	−0.4554 to 0.1535
Alcohol	−0.0672	0.6724	−0.3717 to 0.2504	−0.1505	0.3414	−0.4421 to 0.1698
Sleep	0.2068	0.1889	−0.1128 to 0.4876	0.1186	0.4546	−0.2012 to 0.4155
Behavior	−0.1382	0.3828	−0.4319 to 0.1820	0.4753	0.0015	0.1914–0.6858
Insight	−0.1127	0.4772	−0.4106 to 0.2069	0.1523	0.3357	−0.1680 to 0.4435
Career	−0.0618	0.6973	−0.3671 to 0.2554	0.2584	0.0985	−0.05865 to 0.528
Total score	−0.0275	0.8626	−0.3370 to 0.2873	−0.0022	0.9887	−0.3144 to 0.3103

WHR, Waist-to-hip ratio; WHtR, Waist-to-height ratio; ∑SF, Sum of skinfolds; BMI, Body mass index; 95%CI, confidence interval.

## 4 Discussion

The SOC police officers from the Espírito Santo Special Missions Battalion (SMB), Brazil, perform highly specialized tactical tasks that require elevated levels of physical preparedness. Their occupational demands are closely associated with physical fitness and lifestyle factors necessary to ensure optimal operational performance (27). Accordingly, the present study aimed to evaluate musculoskeletal discomfort and lifestyle indicators in this elite population under different uniform conditions.

Police officers routinely wear protective equipment required for operational safety, with loads ranging from approximately 10 to 55 kg depending on the mission profile (3–8). Although essential for protection, this load may negatively affect musculoskeletal health and physical performance (12, 14, 15, 28, 29). Therefore, understanding the impact of these conditions on discomfort and lifestyle is of practical relevance.

In the present study, all participants were male, which is consistent with previous investigations involving elite police units (22, 25). This finding reflects the current composition of the unit rather than formal recruitment restrictions based on sex. Although lower female representation has been reported in operational roles, official recruitment processes do not explicitly restrict participation by sex; thus, this imbalance should be interpreted with caution.

Regarding anthropometric indicators, most participants were classified within normal or moderate cardiovascular risk categories based on waist circumference and the waist-to-hip ratio. The observed associations between central adiposity indicators (WC and WHtR) and musculoskeletal discomfort are consistent with previous findings, suggesting that increased abdominal mass may contribute to mechanical overload, particularly in the lumbar region (27, 30–32). However, these associations were weak and should be interpreted cautiously given the cross-sectional design.

Although a high prevalence of overweight was observed based on BMI classification, body fat percentage values indicated a generally favorable body composition profile. This apparent discrepancy may be explained by increased lean mass in this population, highlighting the limitation of BMI as an isolated indicator in physically trained individuals. These findings are consistent with previous studies in tactical populations (21, 33–36).

Another important finding of the present study is the high level of physical activity observed among participants. In contrast to studies reporting sedentary behavior in general police populations (35), the officers in this study were classified as physically active, with weekly physical activity levels substantially exceeding the minimum recommended by the World Health Organization (21–24). The mean weekly physical activity time (875.2 ± 662.8 min) may be explained by the inclusion of structured physical training during working hours, which is a common practice in elite tactical units.

However, these findings should be interpreted within the specific context of a highly specialized unit and should not be generalized to the broader police population. Although high levels of physical activity may contribute positively to operational readiness, reduce injury risk, and enhance performance, further studies are required to confirm these associations.

In addition, population-based data from Vitória (Espírito Santo, Brazil) indicate that only 53.3% of individuals achieve recommended levels of physical activity, while 31.8% are insufficiently active and 10.6% are inactive (37). This contrast highlights the distinct profile of elite police units. Regular physical activity has been associated with multiple health benefits, including reduced occupational stress, improved mental health, enhanced cognitive performance, and better physical conditioning (38).

Regarding lifestyle, SOC officers were predominantly classified as having “good” or “very good” lifestyle profiles, corroborating previous findings from our research group (8, 22, 23). However, it is important to consider that policing is recognized as a highly stressful occupation, characterized by frequent exposure to violence, conflict, and psychological strain (39–41). These factors may negatively impact health and well-being despite overall favorable lifestyle indicators.

Notably, the lowest scores were observed in the work domain, suggesting reduced job satisfaction, as previously reported (8, 22, 23, 42). Occupational stress and burnout are common among police officers and may manifest through psychological, physical, and behavioral symptoms. Although sleep scores were relatively high, low scores in behavioral domains may reflect irritability and stress-related responses. The observed association between behavior and years of service suggests that occupational exposure may influence long-term behavioral patterns; however, further investigation is warranted.

The use of the SOU increased body mass by approximately 15.67%, reflecting the additional load imposed by PPE. This equipment includes ballistic vests, tactical belts, holsters, weapons, and communication devices. Although necessary for protection, previous studies have shown that PPE may impair mobility and increase physiological demand during operational tasks (12, 29, 43).

The increased load may reduce mobility during both aerobic and anaerobic activities, potentially increasing occupational risk. Although mobility was not directly assessed in this study, the additional load may negatively influence performance in tasks requiring agility and high-intensity effort, contributing to musculoskeletal discomfort and potential injury.

Several factors may contribute to musculoskeletal injuries in police officers, including prolonged working hours, physical overload, inadequate planning, improper equipment use, and individual characteristics such as body composition and fitness level (27, 44, 45). In the present study, the use of the SOU was associated with a 35.8% increase in perceived musculoskeletal discomfort, affecting all evaluated body regions.

The highest discomfort levels were observed in the cervical, thoracic, and lumbar regions, as well as in the shoulders, which is consistent with previous findings indicating a high prevalence of spinal pain among police officers (46–49). Prolonged standing, vehicle use, and load carriage are key contributors to this pattern.

In addition, exposure to vibration and prolonged sitting may impair spinal biomechanics, potentially reducing intervertebral disc function (50). Proper load distribution and ergonomic adjustments, such as the use of alternative equipment configurations (e.g., leg holsters), may help mitigate lumbar overload.

Although no significant association was found between physical activity and discomfort, regular exercise remains an important strategy for pain prevention and functional improvement (51). However, further studies are needed to better understand these relationships.

This study has several limitations that should be acknowledged, including its cross-sectional design, reliance on self-reported measures, relatively small sample size, and the inclusion of only male participants from a specialized unit, which limits generalizability. In addition, objective measures of physical activity and musculoskeletal outcomes were not used.

Future research should explore different PPE configurations, intervention strategies, and broader populations while controlling for potential confounders such as prior injuries, fitness levels, and task-specific demands.

In conclusion, the use of the special operations uniform was associated with increased musculoskeletal discomfort across all evaluated body regions, particularly in the lumbar spine. Despite high levels of physical activity and generally favorable lifestyle indicators, the additional load imposed by PPE represents a significant ergonomic challenge. Furthermore, BMI alone may not adequately reflect body composition in this population due to elevated lean mass.

## Data Availability

The raw data supporting the conclusions of this article will be made available by the authors under reasonable request to corresponding author.
